# Magnetic Resonance‐Guided Laser Interstitial Thermal Therapy Using Dual‐Wavelength Dual‐Output Laser Within Two Probe Trajectories for Treatment of Drug‐Resistant Epilepsy

**DOI:** 10.1111/cns.70118

**Published:** 2024-11-21

**Authors:** Jingquan Lin, Wei Gao, Yuqi Ying, Ahmed Abdulsalam Ali Bakrbaldawi, Zhoule Zhu, Chengwei Cai, Xinxia Guo, Jianmin Zhang, Junming Zhu

**Affiliations:** ^1^ Department of Neurosurgery, the Second Affiliated Hospital Zhejiang University School of Medicine Hangzhou China; ^2^ Epilepsy Center, the Second Affiliated Hospital Zhejiang University School of Medicine Hangzhou China; ^3^ Brain Research Institute Zhejiang University Hangzhou China; ^4^ Key Laboratory of Precise Treatment and Clinical Translational Research of Neurological Diseases of Zhejiang Province Hangzhou China; ^5^ Clinical Research Center for Neurological Diseases of Zhejiang Province Hangzhou China; ^6^ State Key Laboratory of Transvascular Implantation Devices Hangzhou China; ^7^ Department of Neurosurgery, the Fourth Affiliated Hospital, International Institutes of Medicine Zhejiang University School of Medicine Yiwu China

**Keywords:** Epilepsy, intraoperative MRI, laser ablation, laser interstitial thermal therapy, seizure

## Abstract

**Objective:**

Magnetic resonance‐guided laser interstitial thermal therapy (MRgLITT) is a novel tool and a minimally invasive treatment to drug‐resistant epilepsy (DRE). The focus of this research was to evaluate the effectiveness and safety of the newly developed dual‐wavelength dual‐output MRgLITT system LaserRO within two probe trajectories in treating DRE patients.

**Methods:**

This is a retrospective analysis conducted at a single center, examining patients with DRE who received treatment with the LaserRO MRgLITT system. The system utilizes a sophisticated laser technology that can be configured as conventional single output for single wavelength or innovative dual outputs for dual wavelengths. The study involved a comprehensive review of patient information, encompassing demographics, seizure history, details related to the surgical parameters, and the subsequent clinical results. Primary outcome was post‐operation seizure outcome defined as Engel Scale Class at the end of follow‐up time.

**Results:**

This study included a total of eight DRE patients received MRgLITT surgery between August 2022 and October 2023. Out of these, there were four mesial temporal lobe epilepsy (MTLE), three focal cortical dysplasia (FCD), and one cavernous malformation (CM) patients. Within the two probe trajectories, seven patients had single wavelength (980 or 1064 nm) laser treatment and one patient had dual‐wavelength (980 and 1064 nm) laser treatment. The median age of the patients was 27 (22–31) years, with a median follow‐up period of 9.7 (8.4–12.1) months. The mean BMI was recorded at 20.24 ± 2.95 kg/m^2^, and epilepsy history was 13 ± 6 years. The median intraoperative blood loss was 5 (5–9) mL, operation time was 231 (169–254) minutes, and length of stay (LOS) was 3 (3–5) days. The mean ablation volume ratio was 96.52% ± 3.67%. In terms of outcomes, over a median follow‐up time of 9.7 (range 8.4–12.1) months, there were two patients got Engel I, five patients got seizure‐free, and one patient decreased 75% seizure. Importantly, no serious complications following the procedures occurred.

**Conclusions:**

The preliminary results indicate that the MRgLITT procedure, which operates dual‐output laser with single or dual wavelengths (980/1064 nm) within the two trajectories, is both effective and safe as a minimally invasive approach for different types of DRE patients.

## Graphical abstract

The newly developed dual‐wavelength dual‐output MRgLITT system LaserRO within two probe trajectories in treating drug‐resistant epilepsy patients.

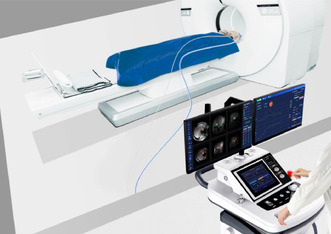

## 1 Introduction

Epilepsy is a chronic brain disorder caused by a variety of factors, characterized by excessive firing of brain neurons, resulting in recurrent, paroxysmal, and transitory central nervous system dysfunction. Epilepsy affects people of all ages, races, and geographies, while its prevalence is higher in children and teenagers [1]. Over 65 million individuals worldwide suffer from epilepsy [2] and the estimated number of individuals with epilepsy is 10 million in China [3]. This chronic neurological disorder not only has a negative long‐term impact on people's quality of life, but it also places a significant load on healthcare systems and families.

At present, the common therapeutic approaches can be divided into: (1) drug treatment; (2) epilepsy surgery (including neuromodulation therapy); (3) ketogenic diet. Among them, surgical resection of epileptic lesions had been recommended for drug resistance epilepsy, which affects around one‐third of patients with epilepsy [4]. While classical craniotomy for epileptogenic lesion resection carries substantial risks and numerous complications. Magnetic resonance‐guided laser interstitial thermal therapy (MRgLITT) has gained popularity as a minimally invasive alternative to surgical resections since FDA approval in 2007 [5]. Compared with the open surgery, MRgLITT is link to reduced index hospitalization expenses, a shorter length of stay (LOS), and a higher likelihood of discharge home [6]. Over the past 10 years, there has been an exponential increase in the application of MRgLITT for DRE in numerous epilepsy center [6, 7, 8, 9, 10].

However, there have been few reports on the use of a dual‐wavelength dual‐output within two probe trajectories to treat DRE patients. This retrospective study aimed to evaluate the effectiveness and safety of the newly designed dual‐wavelength dual‐output MRgLITT system LaserRO in treating DRE at our epilepsy center.

## 2 Methods

### 2.1 Study design

Each admitted patient comprehended and agreed with the informed consent. Retrospective evaluation was conducted on DRE patients treated with MRgLITT utilizing a surgical laser ablation system between August 2022 and October 2023. Every procedure was carried out at our hospital. The research ethics review committee at our hospital approved this study. Prior to the procedure, informed consent forms were signed by each patient or their legal guardian. Data on outcomes, intraoperative procedures, and demographics were gathered and analyzed.

### 2.2 Patient selection

Preoperative evaluations were comprehensive and included 3.0‐T magnetic resonance imaging (MRI) epilepsy imaging protocol and scalp video electrography (VEEG) monitoring. As supplemental testing, positron emission tomography‐computed tomography (PET‐CT) were also employed. In order to assist in locating the epileptic foci, some patients also had electrode installation for invasive EEG monitoring and stereotactic electroencephalography (SEEG). After all data collecting was finished, neurologists and functional neurosurgeons at epilepsy center in our hospital reviewed all of the results after this evaluation to decide on the best way to treat seizures. We selected the DRE patients who received MRgLITT using two trajectories laser for treatment to further analysis in our hospital.

The MRgLITT inclusion criteria were as follows:

1. From the age of 2 to 70 years
2. Drug‐resistant epilepsy diagnosis
3. Focal seizure is the form of epilepsy
4. The epileptogenic focus is confined and appropriate for surgical intervention
5. Informed permission papers were completed and signed by the patients and their families.

The MRgLITT exclusion criteria were as follows:

1. MRI and craniotomy contraindications
2. Epilepsy medication has changed within a month
3. Progressive neurological diseases
4. Sever coagulation disorders
5. Acute infection or chronic infection;
6. pregnant or nursing women

### 2.3 Surgical process

Prior to the procedure, the target position, size, and optic fiber trajectory were planned using contrast‐enhanced MRI in order to produce temporary target coordinates and avoid significant vessels.

**FIGURE 1:**
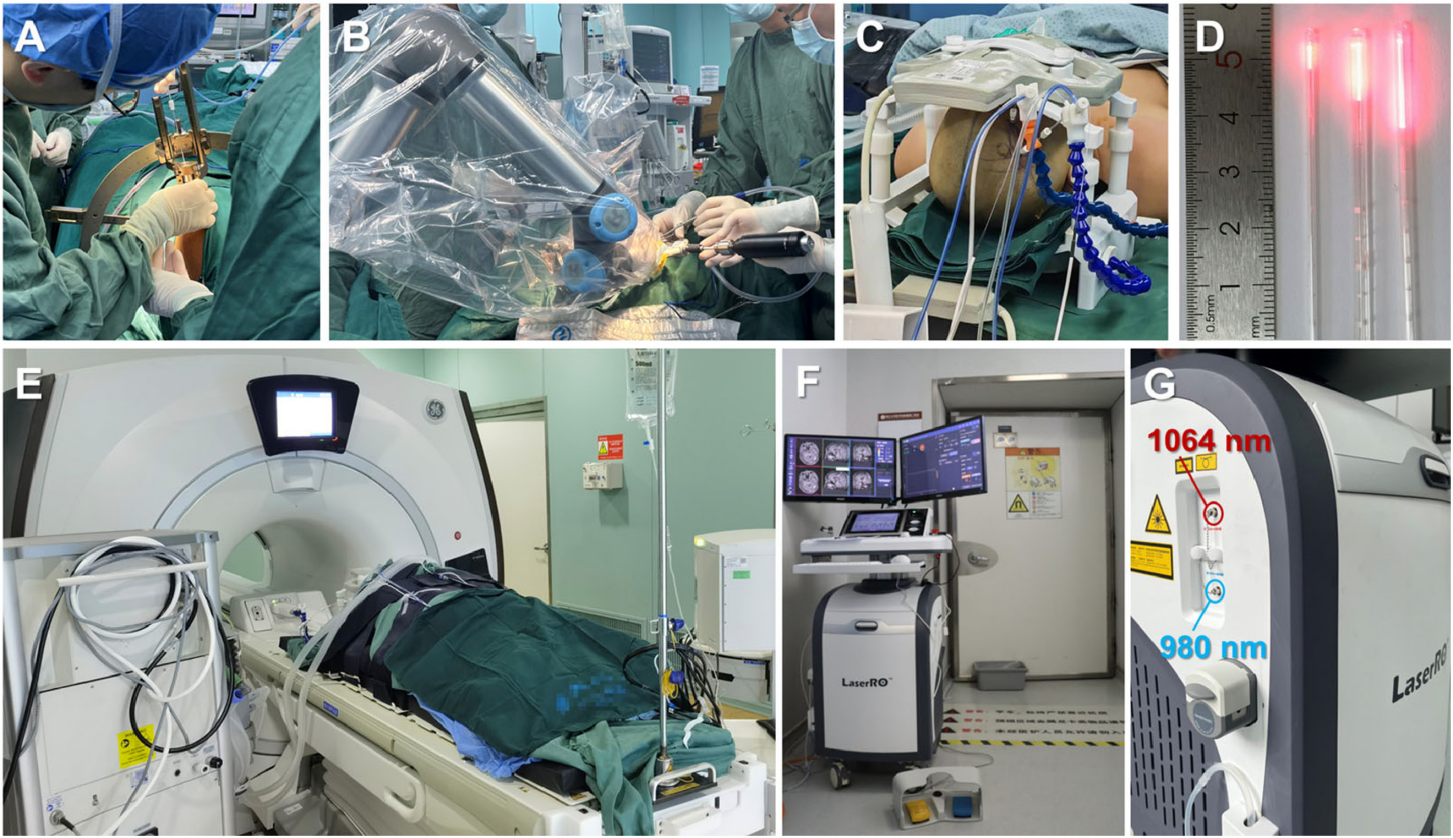
LITT procedure was performed in the intraoperative MRI room. (A) Optical fiber implantation under Leksell frame; (B) neurosurgical robot assisted optical fiber insertion; (C) intraoperative MR coil fixation after optical fiber insertion; (D) Fiber‐optic catheter outer (length 4, 10, 15 mm); (E) patients in the intraoperative MRI room; (F, G) the LaserRO system equipped with a dual‐wavelength laser, operating at 980 and 1064 nm, for MRgLITT.

All the surgical process were performed after general anesthesia. Either the Leksell stereotactic frame or the Remebot neurosurgical robot (NOVOLIFE, Shandong, China) were used for stereotaxy. The target position, size, and optic fiber trajectory were preoperatively planned using Stereotactic Surgery Planning Software (V1.0, Genlight, Hangzhou, Zhejiang, China) to producing temporary target coordinates. The skin was cut and the skull was drilled by electric drill. Then the dura was burned by electrocoagulation. Fix the skull bone anchor, calculate the depth of electrode insertion, and after the hard cleaning rod breaks into the brain to build a channel, insert and fix two laser optical fiber in sequence. The patient then underwent an initial intraoperative MRI (Discovery MR750w 3.0T, GE Medical System, US) scan to check the accurate positioning of optical fiber (Figure 1).

**FIGURE 2:**
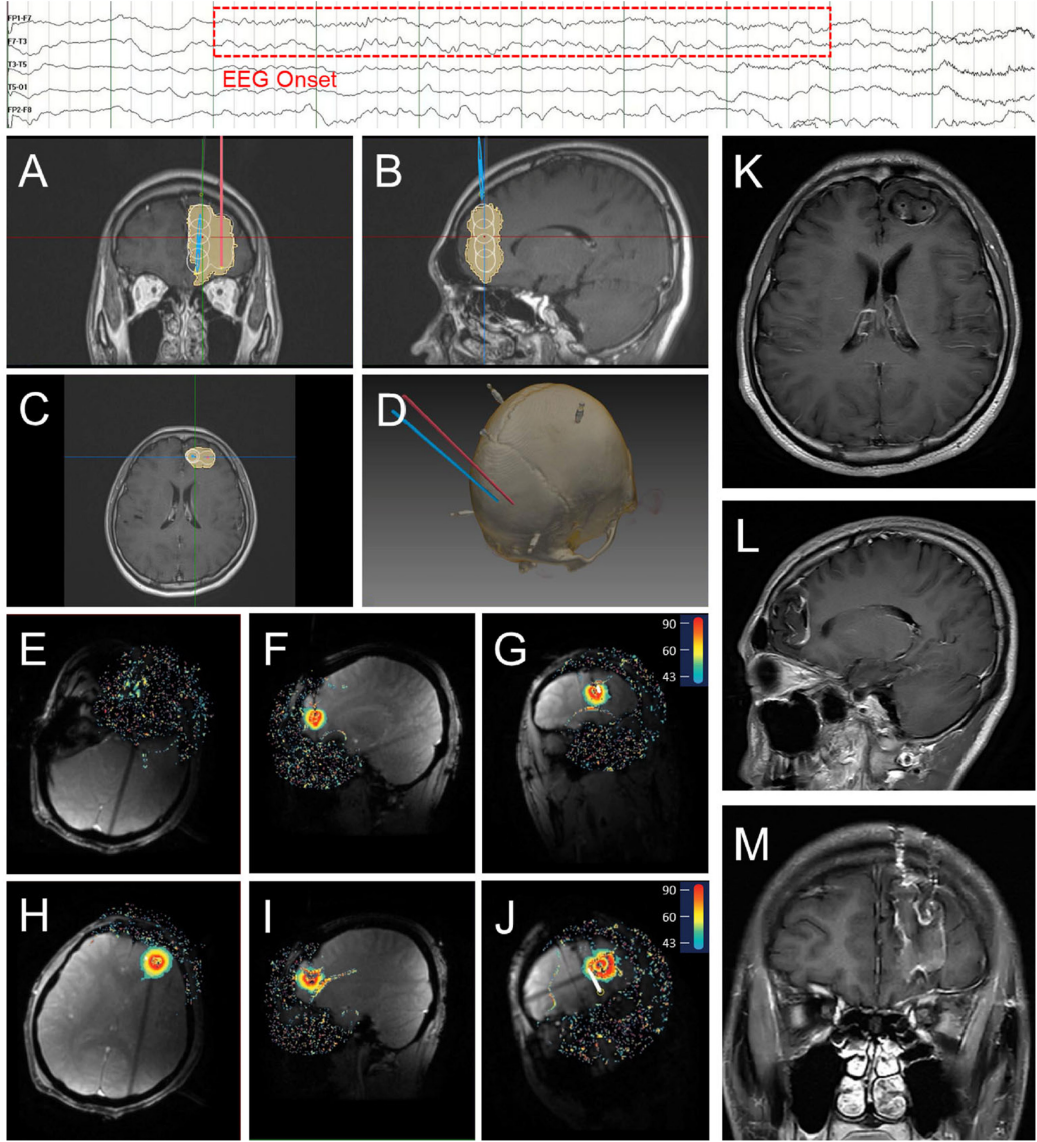
Representative FCD patient's preoperative ablation trajectory planning, intraoperative ablation real‐time thermal monitoring, and postoperative MRI. The top of the figure indicates EEG onset. (A–C) Ablation trajectory planning in coronal, sagittal, and axial plane. (White indicates the planned ablation and yellow indicates the actual ablation); (D) thin‐slice head CT with reconstruction; (E–G) ablation along the first trajectory in axial, sagittal, and coronal plane; (H–J) ablation along the second trajectory in axial, sagittal, and coronal plane; (K–M) postoperative 3‐month MRI in axial, sagittal, and coronal plane.

**FIGURE 3:**
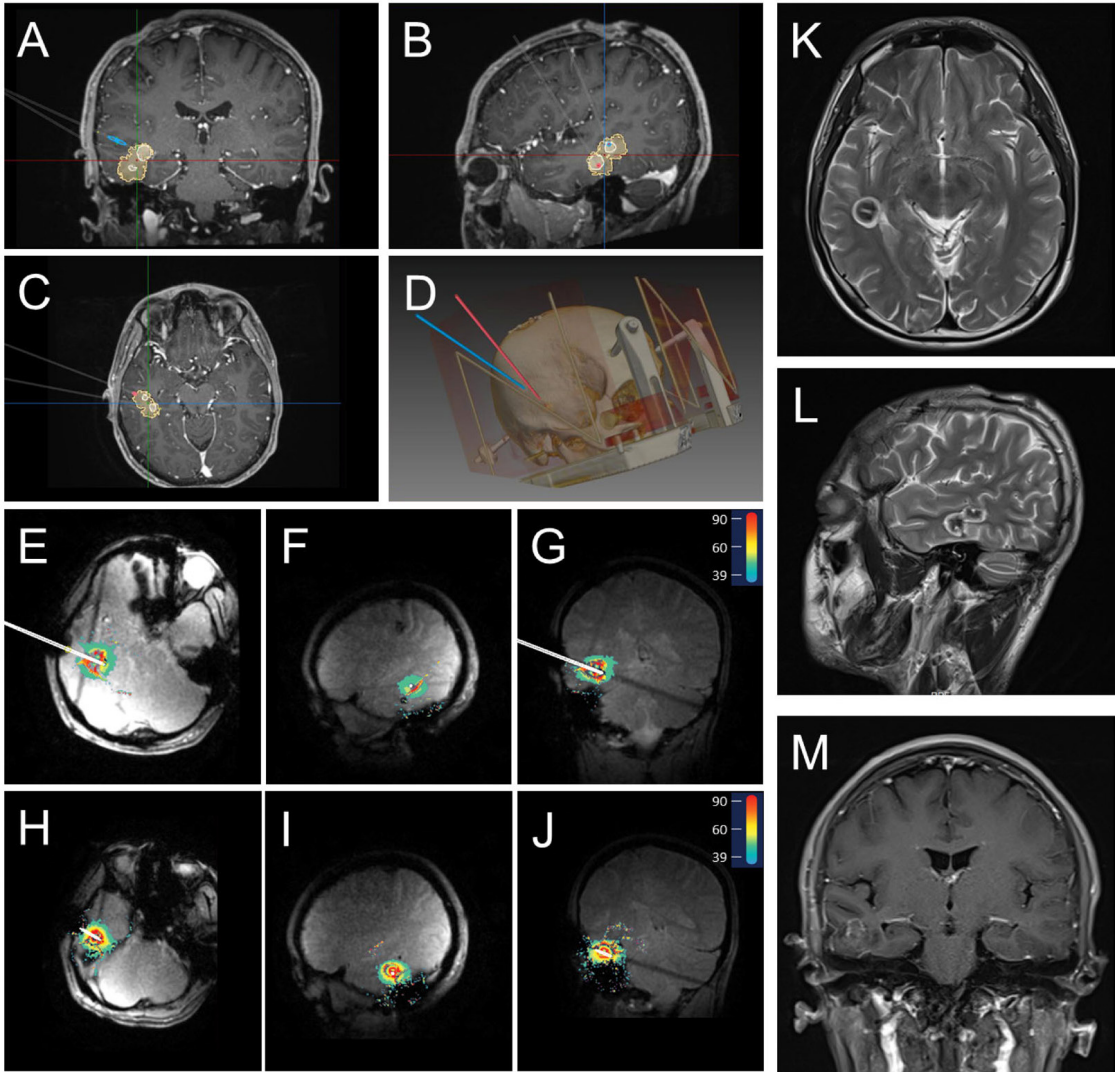
Representative CM patient's preoperative ablation trajectory planning, intraoperative ablation real‐time thermal monitoring, and postoperative MRI. This patient shows no typical EEG onset. (A–C) Ablation trajectory planning in coronal, sagittal, and axial plane. (White indicates the planned ablation and yellow indicates the actual ablation); (D) thin‐slice head CT with reconstruction; (E–G) ablation along the first trajectory in axial, sagittal, and coronal plane; (H–J) ablation along the second trajectory in axial, sagittal, and coronal plane; (K–M) postoperative 3‐month MRI in axial, sagittal, and coronal plane.

**FIGURE 4:**
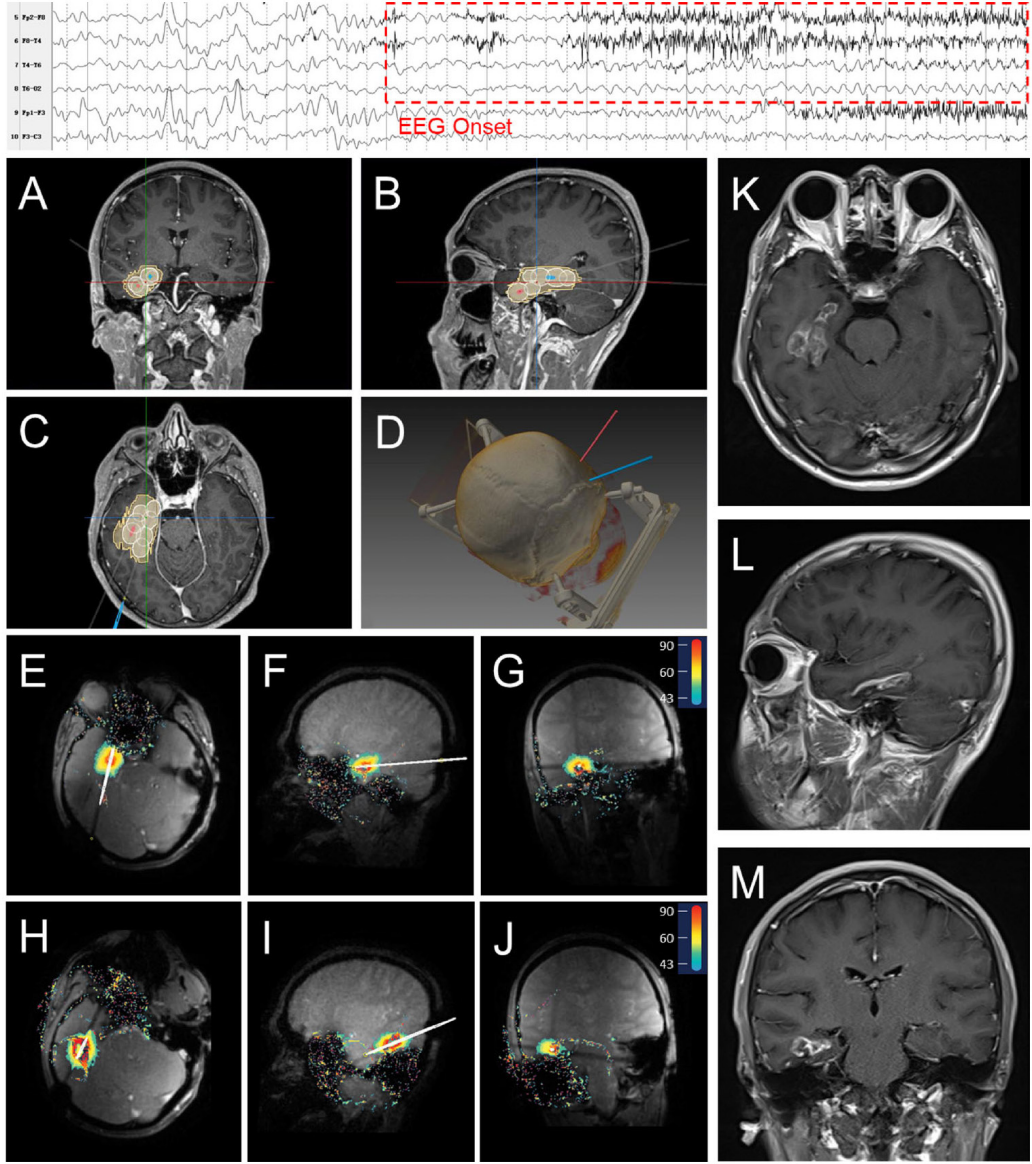
Representative MTLE patient's preoperative ablation trajectory planning, intraoperative ablation real‐time thermal monitoring, and postoperative MRI. The top of the figure indicates EEG onset. (A–C) Ablation trajectory planning in coronal, sagittal, and axial plane. (White indicates the planned ablation and yellow indicates the actual ablation); (D): thin‐slice head CT with reconstruction; (E–G) ablation along the first trajectory in axial, sagittal, and coronal plane; (H–J) ablation along the second trajectory in axial, sagittal, and coronal plane; (K–M) postoperative 3‐month MRI in axial, sagittal, and coronal plane.

All ablations were performed using the LaserRO MRI‐guided laser ablation system (Genlight Inc. Hangzhou, Zhejiang, China), comprises preoperative planning and intraoperative ablation. Preoperative planning involves inputting the ablation surgical plan (target and trajectory information, ablation parameter planning) into the surgical platform. Intraoperative procedure is based on the ablation platform, fiber kit and surgical tools, using real‐time images from the MR equipment to perform the treatment. The system outputs cooling water to the fiber probe through the cooling module. Throughout the treatment process, the ablation platform acquires real‐time images to detect the temperature of the target and its surrounding tissues to ensure safe and effective ablation (Figures 2, 3, 4).

The LaserRO system has unique technical specifications in its design. In terms of main performance, it employs dual‐wavelength light sources of 980 and 1064 nm, with a maximum laser power of up to 15 W. The probe comes in two outer diameters: 1.55 and 2.3 mm, with three different diffusing tip lengths: 4, 10, and 15 mm. The cooling system provides real‐time flow and residual monitoring. The inserted fiber probe is compatible with 1.5 and 3.0T MRI environments, with a 3.5‐second MRI temperature detection interval and a temperature measurement accuracy of ±2°C.

After the ablation process, an MRI scan was used to confirm the ablation range. The probe and skull bone anchor were then withdrawn, and the incision was closed with sutures. The patient was transported to the general ward for overnight observation, during which we routinely did a postoperative CT scan to detect hemorrhage.

### 2.4 Statistical analysis

The Shapiro–Wilk method was used to assess data in this investigation for normality test. Continuous variables were defined as mean ± standard deviation (SD) or median (interquartile range). Categorical variables were expressed as numbers (*n*) and percentages (%). The analyses were performed using SPSS version 25.0 (IBM Co., Armonk, NY).

## 3 Results

### 3.1 Demographic Data

From August 2022 and October 2023, eight patients at our hospital had MRgLITT surgery using two trajectories optical laser. The median age of the operation age was 27 (22–31) years, with 6 male (75%) and 2 female (25%). The mean BMI was recorded at 20.24 ± 2.95 kg/m^2^, and epilepsy history was 13 ± 6 years. There are four patients with mesial temporal lobe epilepsy (MTLE), three with focal cortical dysplasia (FCD), and one with a cavernous malformation (CM). Within the two trajectories, seven patients received single wavelength (980 or 1064 nm) laser treatment, while one received dual‐wavelength (980 and 1064 nm) laser treatment (Tables 1 and 2).

**TABLE 1:** Drug‐resistant epilepsy patient data.

No.	Sex	Age (years)	BMI (kg/m^2^)	DRE	Seizure histories (years)	Laser wavelength (nm)	Blood loss (mL)	Operation time (min)	Hospital stays (days)	Ablation volume rate (%)	Follow‐up time (months)	Engel scale class	AEDs
1	M	27	16.53	FCD	24	980 & 1064	5	400	2	90.33	22.6	I	LEV, CBZ
2	F	30	21.63	MTLE	14	1064	10	250	3	92.05	12.4	I	OXC, LCM
3	F	31	20.31	MTLE	8	1064	10	210	3	99.73	11.4	Seizure free^a^	LCM
4	M	27	16.90	CM	4	980	5	155	3	98.37	10.0	Seizure free^a^	OXC, LCM
5	M	32	25.20	MTLE	11	1064	5	155	3	94.82	9.3	Decrease 75%^a^	OXC, VPA, PB
6	M	15	21.33	FCD	10	1064	5	250	5	99.41	9.1	Seizure free^a^	OXC, LEV, PER
7	M	27	22.04	FCD	19	1064	5	212	5	99.59	8.2	Seizure free^a^	CBZ, VPA
8	M	30	17.96	MTLE	10	1064	5	255	6	97.84	8.2	Seizure free^a^	OXC, LEV

Abbreviations: AED, antiepileptic drugs; CBZ, carbamazepine; CM, cavernous malformation; DRE, drug‐resistant epilepsy; F, female; FCD, focal cortical dysplasia; LCM, lacosamide; LEV, levetiracetam; M, male; MTLE, mesial temporal epilepsy; OXC, oxcarbazepine; PB, phenobarbital; PER, perampanel; VPA, valproic acid.

^a^ Follow‐up time less than 12 months.

**TABLE 2:** Summary of demographic and outcome data.

Patient	Data
Number	MTLE 4; FCD3; CM 1
Age (years)	27 (22–31)
Sex	Female 2; Male 6
BMI (kg/m^2^)	20.24 + 2.95
Seizure histories (yeas)	13 ± 6
Blood loss (mL)	5 (5–9)
Operation time (min)	231 (169–254)
Hospital stays (days)	3 (3–5)
Ablation volume rate (%)	96.52 ± 3.67
Follow‐up time (months)	9.7 (8.4–12.1)
Outcome	
Engel I(*n*)	2
Seizure free (*n*)	5
Decrease 75% (*n*)	1

Abbreviations: CM, cavernous malformation; FCD, focal cortical dysplasia; MTLE, mesial temporal epilepsy.

### 3.2 Perioperative and outcome data

The median intraoperative blood loss was 5 (5–9) mL, operation time was 231 (169–254) minutes, and length of stay was 3 (3–5) days. The mean ablation volume ratio was 96.52% ± 3.67%. In terms of outcomes, over a median follow‐up time of 9.7 (range 8.4–12.1) months, there are two patients got Engel I, five patients got seizure free, and one patient decreased 75% seizure. Importantly, there were no reports of serious complications such as prolonged edema, hemorrhage, and visual field loss following the procedures (Tables 1 and 2).

## 4 Discussion

With a relative decrease in the use of surgical resections and relative increases in usage of LITT for the treatment of DRE patients [11]. LITT is a both effective and safe approach to treat seizures without necessitating a craniotomy [12, 13, 14]. Our epilepsy center has conducted a preliminary exploration of LITT in the treatment of refractory epilepsy. As shown in Table 1, this study of eight patients with DRE who underwent MRgLITT treatment showed that all the patients had a high rate of postoperative epilepsy control. Seven patients did not experience any seizures and one MTLE patient experienced a considerable decrease in the rate of seizures during the median follow‐up period of 9.7 (8.4–12.1) months. Our preliminary early outcome demonstrated that the efficacy and safety of the newly developed dual‐wavelength dual‐output MRgLITT system LaserRO in treating DRE with no serious complications.

Among our eight DRE patients, there were four MTLE, three FCD, and one CM in our study. MTLE remains the most common type of DRE. The gold standard for MTLE is still anterior temporal lobectomy (ATL), which has been shown to be effective in two Class I trials and has rates of seizure freedom ranging from 60% to 80% after a 2‐year follow‐up [15]. While MRgLITT is becoming a minimally invasive alternative to open craniotomy for the remove of epileptic lesions. As for MTLE, a multicenter cohort study showed Engel I seizure freedom was achieved in 55.8% (149/267) at 1 year and 52.5% (126/240) at 2 years after MRgLITT treatment [7]. Other report has reported that the degree of cluster ablation in the amygdalohippocampal complex was substantially correlated with the seizure outcome following LITT in individuals with MTLE [16]. As for FCD and CM, 63.6% (7/11) children epilepsy patients with FCD underwent LITT achieved Engel Class I [17] and 83.3%(5/6) CM patients got Engel I seizure freedom [18]. In terms of outcomes at our epilepsy center, using dual‐wavelength dual‐output MRgLITT system, over a median follow‐up time of 9.7 (range 8.4–12.1) months, seven patients achieved seizure freedom during follow‐up after the procedure, and one MTLE patient got a considerable seizure decrease. Following the procedures, no serious complications occurred. The higher postoperative epilepsy control rate in our center is may related to the shorter follow‐up time. On the other hands, we applied the dual‐wavelength dual‐output MRgLITT system within the two trajectories may be more beneficial to the damage of lesions. Besides, safety is the main advantage of this minimally invasive surgery, which referring the 5 mL intraoperative blood loss and the 3 days length of stay approximately in our study. There is one reported that approximately 7.26 mL blood loss with average 4.95 days LOS [19] and another study showed the LOS was 1 night for 16 (89%) patients [20].

Our study also showed newly developed dual‐wavelength dual‐output MRgLITT system is feasible for the treatment of DRE without requiring a craniotomy. Different wavelengths laser differs in ablation range, heat transfer, and ablation duration. This MRgLITT system combines two laser wavelengths, 980 and 1064 nm, and can switch freely between the two wavelengths for ablation, making it better suited for lesions of different textures, shapes, and sizes. Besides, such as Figure 2 when the lesion area is large and a single optical fiber cannot meet the needs, dual or even multiple optical fibers need to be inserted. However, the ablation range of dual optical fibers is limited if only the 980 nm wavelength is used. Therefore, introducing 1064 nm wavelength for combined ablation can obtain a better ablation range. In summary, compared with other MRgLITT systems, (1) The dual‐output channel design allows wavelength switching during multi‐segment ablation to achieve better conformity; (2) The dual‐wavelength combination of 980 and 1064 nm can be used for larger lesions, reducing the number of optical fibers placed; (3) When the preoperative plan fails to correctly estimate the ablation range, switching wavelengths can avoid the risk of secondary optical fiber placement.

## 5 Conclusion

MRgLITT is a minimally invasive approach for ablation of DRE lesions with great safety, low complication rate, and satisfactory surgical outcomes, especially using dual‐output laser with single or dual wavelengths (980/1064 nm) within the two trajectories. It could potentially replace craniotomy for some DRE patients in the future. However, given the limitations of this investigation, preliminary small sample size results and short‐term follow‐up, a large sample prospective and multi‐center RCT of MRgLITT study are still needed to provide sufficient and necessary evidence.

## Ethics Statement

Informed consent was obtained from the patient to publish his clinical imaging and data, and approval for this study was provided by the Institutional Review Board of the Second Affiliated Hospital of Zhejiang University School of Medicine (2022‐203).

## Consent

All authors approved the final manuscript and the submission to this journal.

## Conflicts of Interest

The authors declare no conflicts of interest.

## Data Availability

The data that support the findings of this study are available from the corresponding author upon reasonable request.
